# Prefrontal Cortex Activation During Dual Task With Increasing Cognitive Load in Subacute Stroke Patients: A Pilot Study

**DOI:** 10.3389/fnagi.2019.00160

**Published:** 2019-07-02

**Authors:** Eric Hermand, Bertrand Tapie, Olivier Dupuy, Sarah Fraser, Maxence Compagnat, Jean Yves Salle, Jean Christophe Daviet, Anaick Perrochon

**Affiliations:** ^1^Laboratoire HAVAE, EA6310, Université de Limoges, Limoges, France; ^2^Médecine Physique et de Réadaptation, Centre Hospitalier Universitaire, Limoges, France; ^3^Laboratoire Move, EA6314, Poitiers University, Poitiers, France; ^4^Faculty of Health Sciences, Interdisciplinary School of Health Sciences, University of Ottawa, Ottawa, ON, Canada

**Keywords:** functional near-infrared spectroscopy, prefrontal cortex, stroke, dual task, gait, cognition

## Abstract

Stroke patients often exhibit difficulties performing a cognitive task while walking, defined as a dual task (DT). Their prefrontal cortex (PFC) activity is higher in DT than in single task (ST). The effects of an increasing load on PFC activity during DT in subacute stroke patients remains unexplored. Our objective was to assess the effects of N-back tasks (low/high load) on cerebral activity, gait parameters, and cognitive performances. Eleven subacute stroke patients (days post-stroke 45.8 ± 31.6) participated in this pilot study (71.4 ± 10 years, BMI 26.7 ± 4.8 kg.m^−2^, Barthel index 81.8 ± 11.0). Patients completed a ST_walk_, and 4 conditions with 1-back (low load) and 2-back (high load): ST_low_, ST_high_, DT_low_, and DT_high_. Overground walking was performed at a comfortable pace and -N-back conditions were carried out verbally. Both gait (speed, stride variability) and cognitive (rate of correct answers) performances were recorded. Changes in PFC oxyhemoglobin (ΔO_2_Hb) and deoxyhemoglobin (ΔHHb) were measured by functional near infrared spectroscopy (fNIRS). Results showed an increase of ΔO_2_Hb while walking, which was not augmented by cognitive loads in DT. Walking speed was reduced by low and high cognitive loads in DT compared to ST_walk_ (*P* < 0.05), but was not different between DT_low_ and DT_high_. Cognitive performances were negatively impacted by both walking (*P* < 0.05) and cognitive load (between “low” and “high,” *P* < 0.001). These data highlight a “ceiling” effect in ΔO_2_Hb levels while walking, leaving no available resources for simultaneous cognitive tasks, during the early recovery period following stroke. In these patients, cognitive, but not motor, performances declined with a higher cognitive load.

## Introduction

Stroke patients with brain lesions may exhibit impaired cognitive functions, altered walking capacity, or both (Grotta, 2016). These declines in cognitive and walking performances are accentuated when they are performed simultaneously in dual task (DT) such as walking while performing a cognitive task (Al-Yahya et al., 2011), which illustrates a cognitive-motor interference in these patients (Plummer et al., 2013).

It is not possible yet to study gait directly in a scanner environment, such as functional magnetic resonance imagery (fMRI), although a few studies proposed that the imaging of ankle dorsi-flexion, a component movement of gait, may provide a useful marker for gait recovery (Johansen-Berg, 2007). Functional near infrared spectroscopy (fNIRS) has proven to be an effective tool for acquiring brain activity during human walking (Perrey, 2014) and in DT (Holtzer et al., 2014; Leone et al., 2017). fNIRS studies that have assessed changes in oxygenated hemoglobin (ΔO_2_Hb) levels in stroke patients, have reported greater changes in the prefrontal cortex (PFC) during DT than in single task (ST) (Al-Yahya et al., 2016; Hawkins et al., 2018; Liu et al., 2018). This change in PFC activation is similar to what has been observed with fMRI in stroke patients performing simulated motor activities (Al-Yahya et al., 2016) and to healthy older adults performing a dual-task in the fMRI scanner (Erickson et al., 2007). This implies that walking in DT requires additional attentional resources. The observed decline in performances during DT are greater in cognition than in gait, suggesting that stroke patients prioritize walking over cognition during DT, contrary to age-matched controls (Mori et al., 2018). These studies included only chronic stroke patients (Al-Yahya et al., 2016; Hawkins et al., 2018; Liu et al., 2018; Mori et al., 2018), in which rehabilitation potentially allowed a recovering of equilibrium reflexes and stepping to a greater extent than patients in subacute phase (Stinear, 2017). Moreover, the effects of DT walking with increasing cognitive load have demonstrated decrements in cognitive performances in healthy older adults (Fraser et al., 2016) but this has yet to be investigated in stroke patients.

In this study, our main objective was to investigate the effects of increasing cognitive load on bilateral, affected and unaffected PFC activation during DT, in subacute stroke patients. The second objective was to assess the cost of DT on gait and cognitive performances and their associations with PFC activation.

## Participants and methods

### Participants

Eleven subacute stroke patients (6 men, 5 women) participated in this pilot study, in the center of Physical Medicine and Rehabilitation (University Hospital, Limoges). Inclusion criteria for post-stroke patients included: acute (<2 weeks after stroke) or early subacute stroke (between 2 weeks and 3 months) (HAS, 2012; Ammann et al., 2014), first stroke located in left or right middle cerebral artery and being able to walk 10 meters with or without assistance (orthotics, crutch) and corrected hearing/vision. Exclusion criteria included previous neurological disease limiting gait, aphasia, pre-existing cognitive disorders (including mild cognitive dementia, Alzheimer and Parkinson diseases), cardiovascular or pulmonary diseases. Functional ambulation category (Holden et al., 1984) for each patient was evaluated on test day, from 0 (non-walking) to 5 (walking alone, stairs included) (FAC, Table 1). Level of education assessed according to the International Standard Classification of Education (Schneider, 2013), from 0 (pre-primary) to 8 (Ph.D. or equivalent).

**Table 1:** Clinical characteristics of patients.

	**Age (years)**	**Level of education (/8)**	**Body mass index (kg.m^**−2**^)**	**Stroke subtype**	**Laterality**	**Days post-stroke**	**Barthel index (/100)**	**Functional ambulation category (/5)**
Pt. 1	60~65	3	37.0	Ischemic	Right	16	100	5
Pt. 2	86~90	3	24.9	Ischemic	Left	16	95	5
Pt. 3	76~80	3	27.6	Ischemic	Left	33	95	3
Pt. 4	56~60	3	26.0	Ischemic	Left	89	75	3
Pt. 5	66~70	5	24.6	Ischemic	Right	98	85	5
Pt. 6	60~65	3	29.2	Ischemic	Right	99	70	3
Pt. 7	66~70	2	31.1	Hemorrhagic	Left	19	85	5
Pt. 8	60~65	/	20.5	Ischemic	Right	22	80	3
Pt. 9	70~75	5	29.4	Ischemic	Right	11	75	5
Pt. 10	76~80	7	21.9	Ischemic	Left	50	70	3
Pt. 11	86~90	3	21.9	Hemorrhagic	Left	48	70	3
Mean ± SD	71.4 ± 10.1	3.7 ± 1.5	26.7 ± 4.8	9 I/2 H	5 R / 6 L	45.5 ± 34.5	81.8 ± 11.0	3.9 ± 1.0

*I, Ischemic; H, Hemorrhagic; R, Right; L, Left*.

The study was approved by national ethic committee (CPP, registration number 2017-A01883-50) and patients gave their informed consent.

### Design Protocol

Patients performed 3 randomly ordered phases successively: cognitive single tasks (i.e., ST_low_ and ST_high_), walking single task (ST_walk_) and DT including simultaneous cognitive and walking conditions (i.e., DT_low_ and DT_high_). Cognitive tasks for ST and DT followed a modified N-back from Fraser et al. (2016) in which the stimuli were presented aurally by the experimenter: “*low*” was associated with the 1-back condition and “*high*” with the 2-back condition. The “*low”* condition was performed before “*high”* condition, separated by a 4–5 min rest. During the N-back test, the experimenter, facing the patient at a distance of 1 m during ST or walking 1 m behind him/her during DT, read aloud and clearly a series of 20 fixed random numbers, between 0 and 10, evenly spaced in a 30-s interval. Responses were recorded with a voice recorder. One practice trial for each cognitive task was conducted prior to experimental testing to ensure proper hearing/vision and a good understanding of each task. In walking conditions, patients walked in an open space at a comfortable pace for 30 s and in DT they were asked to focus equally on walking and cognitive tasks.

### Gait and Cognitive Performances

Patients performed walking conditions (i.e., ST and DT) on an 8-m GAITRite walkway (GAITRite®- CIR Systems, Inc., Sparta, NJ, USA), which provided spatio-temporal gait parameters, such as speed ($\left\|\vec{V}\right\|$, cm.s^−1^), stride variability (tVar, n.u.), and stride asymmetry ($\frac{\text{length of left stride}}{\text{length of right stride}},\text{ n}.\text{u}.$). In cognitive tasks (ST_low_, ST_high_, DT_low_, and DT_high_), the percentage of correct answers was compiled for each condition, as missing or incorrect answers were accounted for as errors. The DT costs on cognition and gait were calculated by:

*Cognitive costs*:

$$\begin{array}{l} \;ccDT_{low}=\;\frac{{Score\;DT}_{low}-\;{Score\;ST}_{low}}{Score\;ST_{low}}\;and\\ ccDT_{high}=\;\frac{{Score\;DT}_{high}-\;{Score\;ST}_{high}}{{Score\;ST}_{high}} \end{array}$$

where Score DT_low/high_ is the cognitive performance (percentage of correct answers); and

*Gait costs*:

$$\begin{array}{l} \;cgDT_{low}\;=\;\frac{\left\|{\vec{V}}_{DT_{\;low}}\|-\;\|{\vec{V}}_{ST_{walk}\;}\right\|}{\left\|{\vec{V}}_{ST_{walk}\;}\right\|}\;and\\ cgDT_{high}\;=\;\frac{\left\|{\vec{V}}_{DT_{\;high}}\|-\;\|{\vec{V}}_{ST_{walk}\;}\right\|}{\left\|{\vec{V}}_{ST_{walk}\;}\right\|} \end{array}$$

The cognitive and gait costs from DT_low_ to DT_high_ are, respectively given by the equations:

$$\begin{array}{l} ccDT_{low\to high}=\;\frac{{Score\;DT}_{high}-\;{Score\;DT}_{low}}{{Score\;DT}_{low}}\;and\\ \;{\text{cgDT}}_{\text{low}\to \text{high}}=\frac{\left\|{\vec{\text{V}}}_{{\text{DT}}_{\text{ high}}}\|-\;\|{\vec{\text{V}}}_{{\text{DT}}_{\text{low}}\;}\right\|}{\left\|{\vec{\text{V}}}_{{\text{DT}}_{\text{low}}\;}\;\right\|} \end{array}$$

### fNIRS Acquisition and Analysis

Cerebral oxygenation was measured using a fNIRS system (Portalite, Artinis Medical, Netherlands). Two optodes were placed on symmetrical prefrontal sites Fp1 and Fp2 according to the EEG 10/20 system. Acquisition was made through the Oxysoft software (version 3.0.97.1). Differential Pathlength Factor was set on 5 as its calculation formula does not apply to patients' age 50 years and older (Duncan et al., 1996). In each condition, after a 30 s rest for baseline, patients performed the 30 s test, before a final 30 s rest phase. A 0.1 Hz low-pass filter was applied to the fNIRS signal to remove physiological and instrumental noise, and motion artifacts were corrected using Matlab-based scripts when needed (Fishburn et al., 2019). The relative concentrations in O_2_Hb and HHb (ΔO_2_Hb and ΔHHb, respectively, μmol.L^−1^) in the test interval (i.e., the last 20 s) were then normalized by subtracting to them the mean value of the last 10 s of baseline, immediately before the beginning of the task, i.e., seated for ST_low_ and ST_high_, and standing for ST_walk_, DT_low_, and DT_high_. From these data were extracted the hemoglobin difference (ΔHbDiff = ΔO_2_Hb − ΔHHb) and the laterality index (LI) defined as the ratio:

$$\begin{array}{l} LI=\;\frac{{\Delta O}_{2}Hb\;\left(affected\;hemisphere\right)\;-\;{\Delta O}_{2}Hb\;\left(unaffected\;hemisphere\right)}{{\Delta O}_{2}Hb\;\left(affected\;hemisphere\right)\;+\;{\Delta O}_{2}Hb\;\left(unaffected\;hemisphere\right)}. \end{array}$$

### Statistical Analysis

A Shapiro-Wilk test confirmed the non-normality of data. Friedman and Wilcoxon tests were then conducted to compare and assess the respective effects of walking (i.e., sit:ST_low_ and ST_high_ vs. walk: ST_walk_, DT_low_, and DT_high_) and cognitive load (i.e., none: ST_walk_ vs. low: ST_low_ and DT_low_ vs. high: ST_high_ and DT_high_) on cerebral activity (ΔO_2_Hb, ΔHHb, ΔHbDiff) and gait parameters (speed, stride variability). A Spearman correlation test was then conducted to establish potential correlations between PFC activity and gait/cognitive performances. The statistical significance was set at *P* < 0.05.

## Results

Individual patients' characteristics and gait/cognitive performances are presented on Tables 1, 2, respectively.

**Table 2:** Gait and cognitive parameters and costs in percentage.

		**ST_**walk**_**	**DT_**low**_**	**DT_**high**_**	**ST_**low**_**	**ST_**high**_**
**GAIT PARAMETERS**						
Speed (cm.s^−1^)		51.6 ± 23.3	41.9 ± 22.0	39.6 ± 20.5		
Cadence (strides.min^−1^)		48.1 ± 24.7	37.5 ± 20.7	40.6 ± 21.3		
Stride duration (s)		0.9 ± 0.3	0.9 ± 0.3	1.0 ± 0.3		
Stride length (cm)		37.6 ± 9.9	33.7 ± 8.9	33.7 ± 9.3		
Stride variability (n.u.)		9.5 ± 6.0	22.3 ± 23.6	18.4 ± 13.9		
Stride asymmetry (n.u.)		1.0 ± 0.3	1.0 ± 0.4	0.9 ± 0.3		
**COGNITION**						
Answer numbers			9.8 ± 3.8	7.2 ± 1.2	9.7 ± 3.0	7.2 ± 1.9
Correct answers			7.6 ± 3.8	2.4 ± 1.7	8.1 ± 3.4	3.8 ± 1.8
**COSTS (%)**						
Cognitive cost (percentage of correct answers)	Cognitive load		DT_low_ → DT_high_ **−58** **±** 31^*^%		ST_low_ → ST_high_ **–**46 ± 25%	
	Gait		ST_low_ → DT_low_ **–**8 ± 36%			
				ST_high_ → DT_high_ **–**27 ± 54%		
Gait cost (speed)		ST_walk_ → DT_low_ **−15** **±** 19^*^%				
		ST_walk_ → DT_high_ **−19** **±** 18^*^%				
			DT_low_ → DT_high_ **–**5 ± 14%			

p significant (0.05) is presented in bold print^*^.

### PFC Activation and Its Correlations With Gait/Cognitive Performance

**Figure 1:**
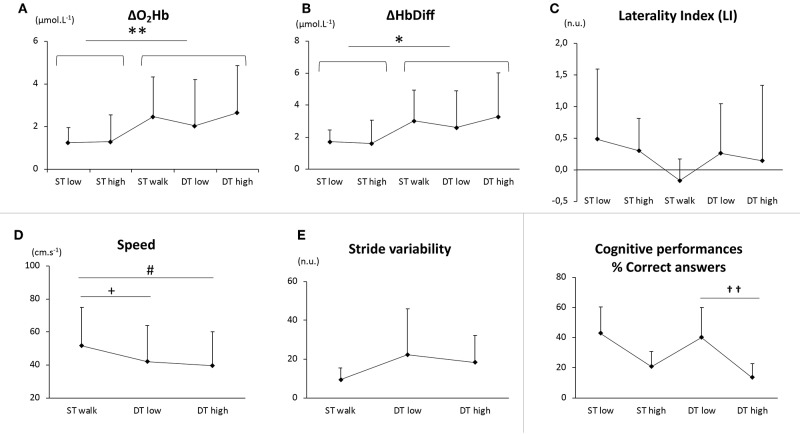
PFC oxygenation values (ΔO_2_Hb, **A**; ΔHbDiff, **B**; laterality index, **C**), gait parameters (speed, **D**; stride variability, **E**), cognitive performances (percentage of correct answers, **F**) and in five conditions: single cognitive tasks at low and high load (ST_low_ and ST_high_), single motor task (ST_walk_) and dual tasks at low and high cognitive load (DT_low_ and DT_high_). On **(A,B)**, the effect of locomotion is illustrated between sit (ST_low_ + ST_high_) and walk (ST_walk_ + DT_low_ + DT_high_) conditions (^*^P < 0.05). On **(D,E)**, differences were noted between ST_walk_ and DT_low_ (^+^*P* < 0.05), and between ST_walk_ and DT_high_ (^#^P<0.05). On **(F)**, differences in “DT_low_” and “DT_high_” cognitive loads were reported for the percentage of correct answers (^††^*P* < 0.01).

There was a main effect of walking with an increase of ΔO_2_Hb (*P* < 0.01, Figure 1A) and ΔHbDiff (*P* < 0.05, Figure 1B) in bilateral PFC, but there was no difference between the different walking conditions (ST_walk_ vs. DT_low_ vs. DT_high_, *P* > 0.05) or between cognitive conditions. No effect of cognitive load was observed for other oxygenation parameters, such as ΔHHb.

Taken separately, we observed a similar increase of ΔO_2_Hb in the respective affected and unaffected hemispheres (*P* < 0.01) whereas LI was not modified (Figure 1C).

Finally, there were no significant correlations between PFC activation (for total, affected or unaffected hemispheres) and gait/cognitive performances (raw values or DT costs, *P* > 0.05).

### ST vs. DT

Speed decreased and gait variability increased in DT_low_ and in DT_high_ compared to ST_walk_ (*P* < 0.05, respectively Figures 1D,E). Walking did not affect the percentage of correct answers (Figure 1F).

The gait cost of cognitive load in “high” condition (i.e., cgDT_high_) was superior to cgDT_low_ (respectively −19 ± 18% and −15 ± 19%) but walking did not significantly influence the cognitive cost (i.e., ccDT_high_ and ccDT_low_).

### DT_low_ vs. DT_high_

No difference in speed or stride variability were found between DT_low_ and DT_high_, Cognitive performances were negatively impacted by cognitive load (percentage of correct answers, *P* < 0.01, Figure 1F).

The cognitive cost of heavier cognitive load during DT (i.e., ccDT_low → *high*_) was −58 ± 31% (*P* < 0.01) but cgDT_low → high_ was negligible.

## Discussion

To our knowledge, this is the first study on DT with an increasing cognitive load in subacute stroke patients. Our first finding relates to the resources required for walking in these patients: ΔO_2_Hb is drastically increased during walking and is not further augmented by any additional cognitive load, low or high. In parallel, we observed a decline in gait performances in DT compared to ST, but no difference between the two DT conditions. As for the cognitive performances, the percentage of correct answers was not decreased by locomotion during DT compared to ST, but was negatively impacted by a higher cognitive load.

Our findings are similar to previous work (Hawkins et al., 2018) in which cerebral oxygenation reached a “ceiling” in chronic stroke patients while walking in ST, leaving no attentional resources for other simultaneous cognitive tasks. This cerebral overactivation triggered by a simple motor task such as walking that PFC activation is consistent with previous work reporting a prioritization of resources on locomotor requirements over cognitive ones in stroke patients, contrary to healthy participants (Mori et al., 2018). Unlike other studies (Al-Yahya et al., 2016; Liu et al., 2018), the cognitive load did not significantly impact cerebral oxygenation (Figure 1A). We assume that this difference could be explained by the difference between subacute and chronic stroke patients. The latter may have recovered a substantial proportion of their walking abilities (i.e., walking speed), therefore leaving some unused cerebral resources for other simultaneous cognitive tasks. Subacute stroke patients, in the early period following stroke, may still be in the cerebral recovery process to regain gait automaticity (Skilbeck et al., 1983), and therefore allocate most of available resources to locomotor needs.

Our data could not highlight a compensatory cerebral reorganization between ipsi- and contralesional hemispheres in ST or DT during the subacute phase: when an increase of O_2_Hb was observed in ST_walk_ or DT, as observed in fMRI research (Al-Yahya et al., 2016), PFC activities were similarly augmented in both affected and unaffected hemispheres, and LI was therefore not modified. These results are consistent with observations in chronic stroke patients (Hawkins et al., 2018), whereas other works show different activities between hemispheres in various motor or cognitive conditions (Liu et al., 2018; Mori et al., 2018). In particular, Liu et al. showed the key role of other brain areas, such as the correlation between unaffected supplementary motor areas (SMA) and gait parameters during DT, in patients with less motor abilities (Liu et al., 2018). Similarly, Mori et al. showed a correlation between motor DT cost and right PFC activation in stroke patients but did not find analogous correlation between brain activation and cognitive performance (Mori et al., 2018). However, they performed a post-analysis showing that ipsilesional PFC activation was also negatively correlated with gait DT cost, implying a prioritization of locomotion over cognition by the affected hemisphere, which we could not support with our data.

The phenomenon of overactivation may be put into perspective with changes in gait parameters: in DT, the simultaneous cognitive tasks were demanded to an already overactivated system, therefore the speed decrease between “none” and “low”/“high” cognitive load was expected (Figure 1D). This is illustrated by the gait cost in “high” condition (cgDT_high_ = −19%), as observed in other studies in stroke patients (Hawkins et al., 2018; Liu et al., 2018) and older participants (Hawkins et al., 2018). However, we did not notice any further gait impairment between “low” and “high” cognitive loads (DT_low_ vs. DT_high_, Figure 1D). This would imply, contrary to ΔO_2_Hb ceiling, the potential existence of a minimal walking speed in participants or patients submitted to a “saturation” of brain activation. Further studies including multiple gradual cognitive loads should be conducted to test this hypothesis.

This is the first study to examine the relationship between brain overactivation and cognitive performance, from “low” to “high” condition during DT in stroke patients. In our study, as expected, cognitive performances were diminished by a higher load during DT, as was previously demonstrated in dual-task walking research with younger and older healthy participants (Fraser et al., 2016). We did not find any correlation between ΔO_2_Hb and cognitive performances. Research with younger participants, found negative correlations between ΔO_2_Hb and cognitive performance during DT, but not ST (Mirelman et al., 2014). This further illustrates the remaining attentional resources in healthy participants (Hawkins et al., 2018), although the reduction in cognitive performances remains greater in older adults (Voelcker-Rehage et al., 2006).

The very limited number of patients included in this study was due to the difficulty of performing both walking and cognitive tasks in subacute phase. In parallel, it is important to note that fNIRS studies during walking show technical and methodological risks which can modify results in study findings if they are not controlled (Vitorio et al., 2017). While the current study used filtering techniques to minimize physiological noise and motion artifact in the signal, we could not control for potential contributions from the scalp to cerebral oxygenation, as this would require a multichannel device with short separation channels. Future research should test the same protocol with short separation channels in order to minimize contributions from the scalp effectively. Also, further studies will need to include a larger sample size and an age-matched control population to confirm the present findings. In addition, existing imaging studies (Johansen-Berg, 2007) have demonstrated the involvement of motor and other areas of the brain that would likely also exhibit changes during ST_walk_ and DT: future studies could provide more information on cerebral changes during dual-task walking with stroke by including the level of cerebral O_2_ in additional regions (i.e., premotor cortex, SMA, primary motor cortex).

In conclusion, our study on subacute stroke patients highlights a “ceiling” in PFC oxygenation that is already reached during walking, requiring most of attentional resources in the early stages after stroke. This partly confirms previous findings that demonstrate large decrements in gait and cognitive performances during DT, regardless of cognitive load, which were not associated with changes in cerebral oxygenation in the PFC.

## Data Availability

All datasets generated for this study are included in the manuscript and/or the supplementary files.

## Ethics Statement

This study was carried out in accordance with the recommendations of national ethic committee (CPP, registration number 2017-A01883-50) with written informed consent from all subjects. All subjects gave written informed consent in accordance with the Declaration of Helsinki.

## Author Contributions

AP and JD: study concept and design. BT and EH: acquisition of data. EH, OD, and AP: analysis and interpretation of data. EH, AP, and JD: drafting of the manuscript. SF, MC, and JS: critical revision of the manuscript for important intellectual content.

### Conflict of Interest Statement

The authors declare that the research was conducted in the absence of any commercial or financial relationships that could be construed as a potential conflict of interest.
